# Bullous Pemphigoid Occurring after Stopping Imatinib Therapy of CML: Is a Continuation of Post-Treatment Follow-Up Needed?

**DOI:** 10.3390/clinpract13050096

**Published:** 2023-09-05

**Authors:** Alexander Yakobson, Ala Eddin Neime, Omar Abu Saleh, Kayed Al Athamen, Walid Shalata

**Affiliations:** 1 The Legacy Heritage Cancer Center and Dr. Larry Norton Institute, Soroka Medical Center, Ben Gurion University, Beer Sheva 84105, Israel; 2 Department of Internal Medicine, Soroka Medical Center & Ben-Gurion University, Beer Sheva 84105, Israel; 3 Dermatology and Venereology, The Emek Medical Centre, Afula 18341, Israel

**Keywords:** bullous pemphigoid, dermato-toxicity, tyrosine kinase inhibitors (TKI’s), imatinib (Gleevec), chronic myeloid leukemia (CML)

## Abstract

Advancements and the use of tyrosine kinase inhibitors (TKIs) have revolutionized the treatment of Chronic Myeloid Leukemia (CML), achieving unprecedented success rates and expanding their applications to various neoplasms. However, the use of TKIs is not without its drawbacks. Skin, gastrointestinal, and central nervous systems are particularly susceptible to adverse effects, including a higher incidence of autoimmune responses in treated individuals. In this report, we present a unique case of bullous pemphigoid, a rare autoimmune disease, which has not been previously associated with TKI therapy as an adverse effect, particularly appearing after discontinuing Imatinib^®^ treatment.

## 1. Introduction

Chronic myeloid leukemia (CML) is a type of leukemia that accounts for approximately 15% of adult leukemias, and 14–24% of all juvenile leukemias [1]. In Western countries, the estimated median age at diagnosis of CML is around 60 years, as determined from data collected in the EUTOS and SIMPLICITY registries. More than 20% of CML patients are aged over 70 years. Conversely, in developing countries with younger populations, the median age at diagnosis is less than 50 years. The annual incidence rate of CML per 100,000 population varies based on the age composition of the respective populations, ranging between 1 and 2 per 100,000 [2]. The prevalence is higher among men, with a ratio of 1.4 men for every 1 woman. The first description of this condition dates back to 1845, when several cases presenting with splenomegaly, anemia, and significant granulocytosis were documented. The Philadelphia chromosome (Ph1), a defining characteristic of this condition is a translocation of chromosomes 9 and 22. As a result of this translocation, the BCR-ABL1 hybrid gene is formed, leading to the production of a constitutively active oncokinase protein. The ABL kinase domain, which spans approximately 300 amino acids, adopts a bilobed conformation consisting of a C-terminal lobe and an N-terminal lobe, with ATP binding occurring within a cleft flanked by these lobes. The flexibility of the C-terminal loop, which contains a “DFG” motif (Asp381-Phe382-Gly383), allows the kinase to transition between an active (open) and inactive (closed) conformation. Common symptoms of CML include night sweats, anemia, and fatigue. Laboratory tests often reveal absolute leukocytosis with a left shift, as well as elevated basophils and eosinophils (present in 90% of cases) [3,4,5].

Patients with CML can be classified into three phases: chronic, accelerated, and blastic. The majority of patients (approximately 85%) are diagnosed while in the chronic phase, which is the earliest and most stable phase of the disease [6].

The introduction and use of tyrosine kinase inhibitors (TKIs) marked a significant advancement in the treatment of CML. In 2001, imatinib (Gleevec^®^) became the first TKI to be approved by the Food and Drug Administration (FDA) for CML treatment. TKIs work by inhibiting specific trans-membrane tyrosine kinase peptide receptors, preventing the activation and phosphorylation of target proteins. This mechanism of action specifically targets the fusion product of CML, known as BCR/ABL on the Philadelphia chromosome [6,7].

Numerous studies have demonstrated the efficacy of TKIs in improving outcomes for CML patients, including long-term progression-free survival and overall survival. TKIs have become a cornerstone in the management of CML, offering significant benefits to patients [6,7].

Although the treatment of CML with TKIs has shown remarkable success, it is important to acknowledge that these drugs are not without adverse effects. Various studies have reported cutaneous side effects associated with TKI therapy. Skin toxicity, for example, has been observed in 11–67% of patients. However, to date, there have been no documented cases of bullous pemphigoid (BP) reported in the literature as a side effect of TKIs [8,9].

BP is a relatively uncommon autoimmune skin disorder characterized by the presence of blisters, urticarial lesions, and intense itching that typically affects older individuals. It is characterized by the development or the formation of sub-epithelial blisters, accompanied by the deposition of immunoglobulins and complement within the basement membrane zone of the epidermis and/or mucous membrane. The classic presentation is a tense bulla measuring usually from 1–3 cm in diameter, which appears on an urticarial, erythematous or non-inflammatory base. The common areas of skin involvement include the trunk, flexures of the extremities, as well as the axillary and inguinal folds [7]. To diagnose BP, a skin biopsy from a blister is performed and examined using hematoxylin and eosin (H&E) staining. Additionally, direct immunofluorescence (DIF) is conducted. DIF is widely regarded as the most sensitive diagnostic test for BP, with over 90% of cases demonstrating linear staining of IgG and/or C3 along the basement membrane zone. In addition, there may be less pronounced linear basement membrane zone staining observed for IgM, IgA, and/or IgE [10,11]. BP is associated with notable morbidity and mortality, and the true burden of the disease is frequently underestimated [11].

The stop imatinib trial (STIM1) has provided evidence linking patients with Philadelphia chromosome-positive leukemias to a higher risk of experiencing cutaneous adverse reactions compared to other sub-types of leukemias. Furthermore, it is believed that the reduction or discontinuation of TKIs can trigger the reactivation of the immune system, potentially leading to an increase in autoimmune responses [12].

To our knowledge, while cutaneous side effects are known to occur with TKI therapy, the development of BP in association with these agents has not yet been described in the literature. In this report, we present, for the first time, a case of severe BP occurring as a late toxicity following the discontinuation of a TKI. This highlights the importance of considering the possibility of autoimmune-related adverse effects even after the cessation of TKI treatment. Monitoring and the follow-up of patients who have received TKIs should be continued well after the end of treatment to detect any potential late-onset toxicities, including autoimmune disorders such as BP.

## 2. Case Study

A 61 year old male with a history of hypertension but no other chronic diseases presented to the emergency room in September 2004 with jaundice. On physical investigation the spleen was enlarged to a span of 16 cm. Laboratory tests revealed leukocytosis with eosinophilia and basophilia, as well as elevated liver enzymes (Table 1).

He was hospitalized for further investigation. Bone marrow biopsy and cytogenetic analysis revealed:

∗ Densely packed marrow.
∗ Myeloid hyperplasia with complete range of maturation.
∗ The biopsy findings were most consistent with CML.
∗ Cytogenetic analysis showed a chromosomal translocation with the karyotype: 46,XY,t(9;22)(q34;q11), known as the Philadelphia Chromosome.

The FISH (Fluorescence In Situ Hybridization) test indicated a translocation of BCR/ABL in 55% of cells, with 80% of cells lacking a third signal.

The PCR (Polymerase Chain Reaction) test for BCR/ABL translocation was positive, confirming the diagnosis of CML.

The patient was initiated onto treatment with imatinib 400 mg once daily, which resulted in a partial response. The dose was increased to 800 mg daily (400 mg 2/day), leading to a complete cytogenetic response, shown by the absence of abnormal chromosomes associated with CML. Additionally, the disease was not detectable by PCR after 20 months of treatment. However, imatinib was temporarily discontinued in October 2005 due to cholelithiasis, and the patient later experienced a relapse in April 2007, leading to the resumption of imatinib treatment.

Over the years, the patient achieved complete remission and received imatinib 400 mg twice daily until August 2013 when the dose was reduced to once daily due to renal dysfunction. Later, in April 2017, the treatment was stopped due to a cerebrovascular accident, diarrhea, worsening renal failure and weakness. Two years after stopping imatinib, the patient developed rapidly enlarging and bursting blisters on multiple areas of the body, and he was referred to the hospital for further investigation. He was found to have bursting blisters on his back, right elbow, lower limbs, chest and right forearm. The biopsies from the chest and the back revealed:

∗ Sub-epidermal blisters.
∗ Interstitial and perivascular mixed cell inflammatory infiltrate composed of numerous neutrophils, eosinophils and mono-nuclear cells in the upper-dermis.

The histologic findings were consistent with sub-epidermal blister disease.

Diagnostic evaluation through direct immunofluorescence (DIF), (Table 2).

**Figure 1:**
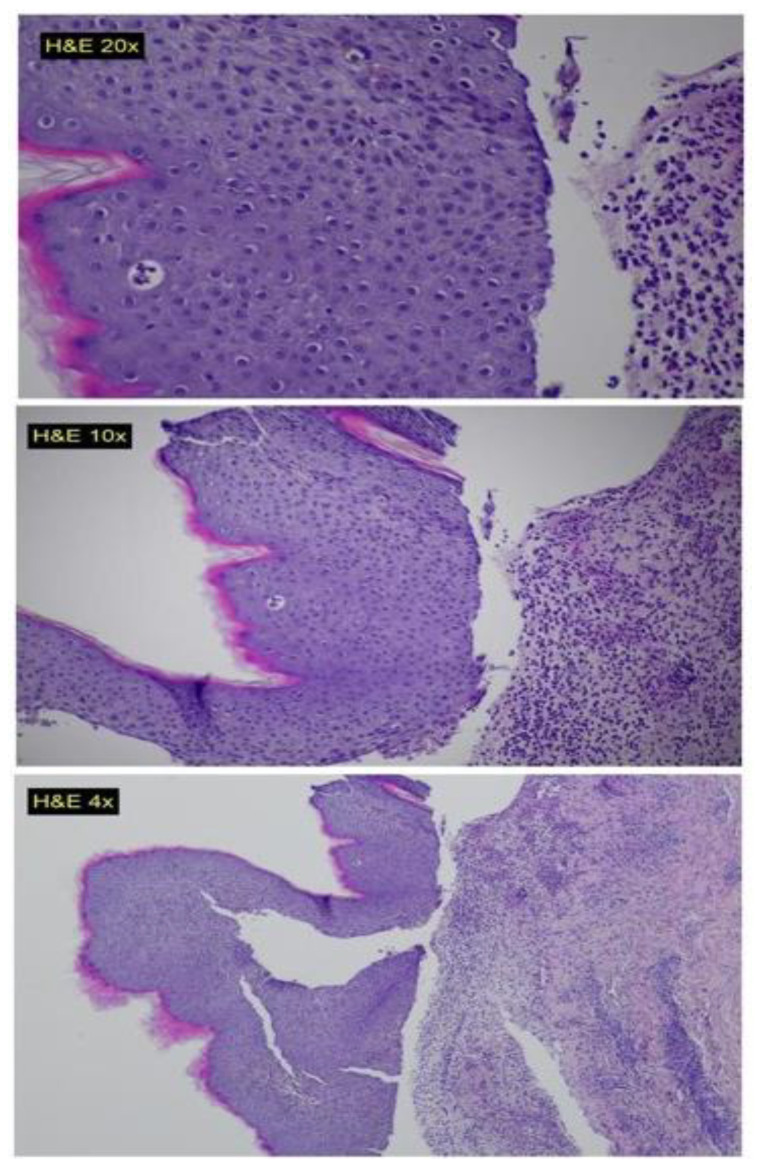
Histopathological slides showing sub-epidermal blisters, with interstitial and perivascular mixed cell inflammatory infiltrate composed of numerous neutrophils, eosinophils and mono-nuclear cells in the upper dermis.

A multidisciplinary team consisting of a hematologist, oncologist, infectious disease specialist, and dermatologist concluded that the patient’s BP was a late toxicity associated with imatinib therapy. This conclusion was based on considering the patient’s overall good health and the absence of any chronic treatments prior to imatinib administration. The patient was treated with antibiotics and topical corticosteroids (oral doxycycline 100 mg twice daily for one month, clobetasol propionate 0.05% cream for the affected areas, 2/day for two weeks) and was discharged home (during his discharge the patient was asked for completing a serology test for BP180 and 230 antibodies, but due to his general and rapid deterioration, it was not performed). After almost three weeks, his condition worsened, and he was readmitted to the hospital. A second biopsy (from the trunk region), (Figure 1) showed sub-epidermal separation and signs of re-epithelization. Unfortunately, there was a noted worsening in the patient’s condition, and he died due to sepsis one week after admission.

## 3. Discussion

We have presented an unusual case of a patient with CML who experienced an extremely rare serious adverse event (AE) as a result of TKI therapy (imatinib^®^), occurring after achieving complete disease response.

Imatinib treatment revolutionized the clinical course and prognosis of CML patients, leading to remarkable advancements in modern medicine. The initial trial involving imatinib, a phase I study, started in 1998 with CML patients categorized as poor risk [13]. Notably, it has demonstrated minimal toxicity while inducing complete cytogenetic remissions (CCyR) in the majority of patients in the chronic-phase of CML. With a median follow-up of 10.9 years, the long-term outcomes have been promising. Among the patients receiving first-line imatinib, the estimated overall survival rate at 10 years was 83.3% (95% CI, 80.1 to 86.6). Nearly half of the patients assigned to imatinib completed the study treatment successfully, and an impressive almost 83% achieved a complete cytogenetic response. BP is not typically associated with TKI use and has not been previously reported in this context [14,15].

BP is frequently observed in patients over the age of 60 and is usually attributed to genetic changes, physical insults, or post-infectious causes, but its occurrence after TKI treatment is unprecedented. In several cases, it was reported as an AE related to immune checkpoint inhibitors therapy [11]. Cutaneous reactions of various types associated with imatinib are not rare, and previous reports indicate a high incidence of such reactions. In several studies, the mean number of cutaneous AEs reported was 1.40 ± 0.95 (per patient) [16]. The primary cutaneous AEs described in relation to imatinib include skin rash, dermatitis, pruritus (itching), vasculitis, edema, erythema nodosum, and various types of eruptions or maculopapular rashes (such as lichenoid and psoriasiform lesions, acute generalized exanthematous pustulosis, and Stevens–Johnson syndrome). The relationship between the occurrence of skin rashes and the dosage of imatinib is not entirely clear. However, studies have indicated that the prevalence of these rashes is highest during the early stages of imatinib treatment. Furthermore, several studies have reported a dose-dependent skin toxicity associated with imatinib, suggesting a pharmacological effect of the drug [17,18,19]. It has been suggested that higher doses of imatinib are more likely to result in skin toxicity and related adverse reactions [17].

The exact cause of these skin rashes remains uncertain. However, it has been observed that the platelet-derived growth factor receptor is abundant in keratinocytes, and the inhibition of this receptor by imatinib may contribute to the development of skin reactions. Other researchers have proposed that certain skin reactions may be a result of the inhibition of KIT, which is present in basal cells. Additionally, it has been noted that hypopigmentation, which is linked to the inhibition of c-kit, may or may not be associated with rashes and is more prevalent in individuals with ethnic pigmentation [17,18,19]. As noted, imatinib therapy may lead to skin rashes, which tend to occur more frequently in women. In many instances, these rashes are mild and localized, often responding well to treatment with antihistamines or topical steroids. However, in severe cases, a short-term course of oral steroids might be necessary. Imatinib associated rashes are typically accompanied by itching. The lesions commonly manifest as erythematous or maculopapular eruptions, primarily affecting the forearms or torso, and less commonly the face. A skin biopsy can often confirm a toxic reaction to the drug [20]. Severe grade 3 or 4 skin rashes are relatively uncommon, occurring in only a small proportion of cases (approximately 3.8%). In certain patients, more serious reactions can manifest, characterized by skin peeling and even the development of Stevens-Johnson syndrome [21]. In such instances, it is recommended to promptly discontinue the therapy and administer systemic steroids. Severe and unresponsive skin lesions have been identified as the most common reason for permanently discontinuing imatinib treatment. It is important to note, however, that the occurrence of this event is rare, affecting less than 1% of all patients [22]. BP has not been previously reported as an adverse reaction to imatinib in the available literature [16,23,24,25,26].

In addition, in the STIM1 trial, it was demonstrated that there was a higher incidence of adverse cutaneous reactions in Ph1-positive leukemia patients treated with imatinib than in other CML subtypes [12]. It was reported also that the cutaneous toxicity can persist even after discontinuation of imatinib. Hypopigmentation, possibly linked to the inhibition of c-kit, may or may not be accompanied by a rash and tends to be more common among patients with ethnically pigmented skin [23]. Additionally, there are some evidences of immune system reactivation in CML patients with good response to TKIs, which means that this reactivation may lead to an auto-immune disease and in some case to a rare disease, as in our patient. We have no other underlying causes or etiologies that were identified to explain the development of BP. Therefore, based on the previous mentioned incidence of cutaneous reactions in Ph-1 positive CML, and on our patient’s good response to the TKI, we can conclude that the adverse effect was induced by imatinib treatment [27]. BP is known to be associated with the remission of symptoms following the resection of solid tumors, as well as the reappearance of lesions with cancer recurrence, which suggests a potential para-neoplastic link. In some cases, there has been a temporal delay with the diagnosis of paraneoplastic BP preceeding the diagnosis of cancer, raising the possibility that immunosuppressive treatment for non-paraneoplastic bullous pemphigoid might be a significant contributing factor to subsequent malignant disease [28].

Rillzabrutinib is a promising compound known for its high potency as a BTK (Bruton’s tyrosine kinase) inhibitor. Unlike irreversible BTK inhibitors, like ibrutinib, rillzabrutinib possesses a unique reversible covalent-binding mechanism. This distinctive feature holds the potential to enhance its safety profile compared to existing inhibitors. It has been approved for hematological diseases, such as chronic lymphocytic lymphoma, small lymphocytic lymphoma and mantle cell lymphoma, and showed to be safe with a rapid clinical activity in pemphigus [29,30].

However, in our case, the patient did not have a solid tumor or any resection. In addition, imatinib is less known to be associated with a second primary tumor and the appearance of BP did not occur until well after the drug was discontinued.

Based on the available information in the literature, to the best of our knowledge, this is the first reported case of fatal BP as an AE in a CML patient after discontinuing imatinib treatment. Our findings underscore the importance of close monitoring and vigilant observation for delayed toxicities in patients with CML and other malignancies following TKI therapy.

We believe that a comprehensive follow-up strategy is essential in the era of targeted therapy, not only for imatinib but also for other available TKIs, to promptly identify and manage potential autoimmune diseases and other AEs.

BP often presents with a severe clinical manifestation, but in many cases, it can be effectively resolved through the use of topical or systemic steroids and discontinuation of the triggering treatment. However, it is crucial to highlight the significance of closely monitoring patients undergoing imatinib therapy or any other therapeutic interventions. Dosage adjustments should be made as necessary to minimize the risks associated with side effects, including those that may pose a fatal threat to the patient. Considering the substantial morbidity and mortality associated with BP, it is imperative for clinicians to be knowledgeable about the common symptoms, laboratory findings, and anticipated onset of this condition following treatment initiation. By being aware of these factors, healthcare professionals can proactively manage and address the condition, thereby improving patient outcomes and minimizing potential complications and even rare fatal events.

## 4. Conclusions

This case highlights the complexity of managing CML and the potential adverse effects associated with long-term treatment with imatinib. The development of BP as a late toxicity underscores the need for post-treatment follow-up and monitoring of patients who have received TKIs to detect and manage potential autoimmune-related complications.

## Figures and Tables

**Table 1:** Patient routine laboratory tests results.

Parameters [Normal Range (Units)]	Results
White blood cells [4.8 to 10.8 (10^3^ cells/μL)]	75
Eosinophil’s [1 to 3%]	5.3%
Basophils [0 to 1.5%]	6.8%
Blasts [%]	0.73
Platelets [130 to 400 (10^3^/μL)]	173
Hemoglobin [14 to 18 (g/dL)]	8.9
Lactate dehydrogenase [150 to 480 (U/L)]	1373
Alanine aminotransferase [0 to 41 (U/L)]	156
Gamma-glutamyl transferase [0 to 49 (U/L)]	295
Aspartate aminotransferase [0 to 37 (U/L)]	76
Total Bilirubin [0 to 1.1 (mg/dL)]	3.4

**Table 2:** Patient’s direct immunofluorescence result that confirmed Bullous Pemphigoid:

IgG	positive + 2 shining—linearly within the region of the basal membrane
IgA	positive + 1 shining—linearly within the region of the basal membrane
IgM	negative
C3	Positive + 4 shining—linearly within the region of the basal membrane
Fibrinogen	Positive + 2 shining—linearly within the region of the basal membrane

## Data Availability

Data are contained within the article or are available from the authors upon reasonable request.
